# Editorial: Clinical hypnosis

**DOI:** 10.3389/fpsyg.2024.1464449

**Published:** 2024-08-22

**Authors:** Ernil Hansen, Burkhard Peter, Thomas G. Wolf

**Affiliations:** ^1^Department of Anesthesiology, University Hospital Regensburg, Regensburg, Germany; ^2^MEG-Foundation, Wilhelmsthal, Germany; ^3^Department of Restorative, Preventive and Pediatric Dentistry, School of Dental Medicine, University of Bern, Bern, Switzerland; ^4^Department of Periodontology and Operative Dentistry, University Medical Center of the Johannes Gutenberg University Mainz, Mainz, Germany

**Keywords:** hypnotherapy, hypnotizability, depression, anxiety, psycho-oncology, chronic pain, irritable bowel syndrome, unconscious

Hypnosis is a powerful and valuable tool in psychotherapy and medicine with a long history. The contemporary form of hypnotherapy mainly developed under the influence of Milton H. Erickson in the last century (Erickson, 1948). Distinct from any authoritarian or esoteric forms it pronounces in the center the patient, not the hypnotherapist; it is a permissive rather than a directive way of working, and a personal relationship instead of a collection of tools. With the patient in the center of the healing process, a unique feature of hypnotherapy at the central role of self-efficacy in the form of self-hypnosis. Some obstacles to a wider application of hypnosis in psychotherapy and psychosomatics and its re-union with medicine are a lack of conclusive studies and the limited access to specialized journals. Hypnosis journals are little known and often accessible only to insiders. More scientific evidence and more articles in scientific journals are needed. Practicing psychotherapists and physicians rarely come in contact with the world of hypnosis (Hansen, 2024). Therefore, the Research Topic “*Clinical Hypnosis*” in this recognized scientific open-access journal is a novel and great step. In 20 articles readers can get—even though without any claim of completeness—an idea of hypnosis and its potential in psychotherapy, psychosomatics, medicine, and dentistry.

## Overview

In an overview, Peter briefly describes the history and development of hypnosis. Its roots can be traced back a long way, in modern times at least around 250 years to the proto-forms of today's psychotherapy. It played an important role in the period of Romantic medicine at the beginning of the 19th century and then again around 1900 in the form of suggestive hypnosis, before it was replaced by psychoanalysis and developments in pharmacology. Peter briefly discusses these periods and then describes the further therapeutic and scientific developments from the middle of the 20th century, when—by today's criteria—serious and still ongoing hypnosis research began, and—particularly through the influence of Milton H. Erickson—contemporary forms of hypnotherapy were developed. His outline of a basic understanding and the clearly scientific and serious representation of hypnosis is—in contrast to the irritating variety of presentations and applications found on the internet and with dubious self-proclaimed hypnosis gurus—particularly suitable for generating interest in the fields of medicine. Another essential key to accessing medicine is the evaluation and explanation of physiological basis of therapeutic techniques, in the case of hypnosis especially findings from electrophysiology and neuroimaging. Miltner et al. review such evidence, specifically the effects of hypnosis on brain functions and structures of chronic pain processing and control. Results of electroencephalography (EEG), magnetoencephalography (MEG), and event-related potentials (ERPs) are presented and discussed, as well as results from magnetic resonance imaging (fMRI) and positron emission tomography (PET). The authors undertake the laudable but difficult challenge to find explanations for the diverse and sometimes contradictory findings, and make valuable methodological suggestions for future research. Finally, the acceptance and application of hypnosis are based on scientific evidence. Accordingly, Rosendahl et al. review the meta-analytic evidence on the efficacy of hypnosis for mental and somatic health issues in the last 20 years. Significant effectiveness is documented for patients with chronic pain, cancer, or irritable bowel syndrome, as well as for painful medical procedures and child birth. Rather sparce evidence had been found for “classical” hypnotherapeutic applications, namely smoking cessation, obesity, somatic complaints and psychosomatic symptoms. This is not due to a lack of treatment efficacy but a lack of studies. Meta-analyses like this are the base for treatment guidelines, and increasingly will reflect “the state of the art” and determine acceptance and application of hypnosis, up to the coverage of treatment costs by health insurance companies. With reported 25% medium and 29% large effects, hypnosis deserves an important role in health care, which is not yet reflected in current use.

## Hypnosis in psychotherapy and psychosomatics

Some examples of current, modern studies on the classical use of hypnosis, namely hypnotherapy, are given in this Research Topic. The article by Haipt et al. describes a special neurophysiological study of the functional connectivity of the default mode network (DMN) in a subgroup of depression patients. These patients had been treated with cognitive behavior therapy or hypnotherapy in a clinical RCT study (Fuhr et al., 2021) proving that hypnotherapy is as effective as the gold standard, cognitive behavior therapy. The present article reports the first study of its kind in which the DMN was investigated over the longer time span of a pre-post measure. It reveals differential effects of the two forms of treatment, which have already been shown by the same group in another study (Haipt et al., 2022). While several studies (see meta-analysis of Milling et al., 2019) indicate that hypnotherapy is an effective treatment for depression, this evidence is still lacking for the major area of anxiety disorders. Here now, the same working group from Tübingen in Germany demonstrates feasibility and effectiveness for this group of disorders in another RCT (Fuhr et al.). As a pilot study, however, it had too few patients to confirm the second hypothesis that hypnotic susceptibility is associated with COMT ^Val108/158^ Met genotype and could predict treatment success for hypnotherapy, and further studies are advised. Batra et al. in the third RCT of this working group, show that hypnotherapy is not inferior to the gold standard cognitive behavior therapy in terms of effectiveness for smoking cessation. After six weekly sessions, 15% of patients stayed absent in the 12-month-follow-up in both treatment groups. With this study, hypnotherapy as a popular treatment for smoking cessation has received a scientific basis.

Hypnotherapy and cognitive behavior therapy were also the methods used in the clinical trial at the University of Ulm in Germany, as reported by Gelse et al.. They studied resource activation for lasting effects on wellbeing and stress management to test psycho-oncology as an integral part of oncology day care. This objective was well confirmed. The interventions each comprised only three individual 1-hour sessions, and treatment effects lasted up to 3 months after intervention. Chronic pain patients are often under opioid treatment, which is why psychological pain therapies are desirable at least as an adjunct. Hypnosis is not only one of the oldest but also one of the most effective methods for this (Peter, 2011; Rosendahl et al.). The team around Ogez et al. has developed a special hypnosis program, Hypnose de la Douleur (HYlaDO) as a self-hypnosis alternative for the treatment of chronic pain following the ORBIT model for designing interventions. Their preliminary evaluation showed significant short-term pain relief, a decrease in anxiety, and increased relaxation after one session, with long-term trends indicating improvements in physical activity and quality of life.

## Hypnosis in medicine and dentistry

A successful and evidenced application of hypnosis in medicine is for irritable bowel syndrome (IBS) as presented in a mini-review by Häuser. This widespread disease affects the brain-gut axis and leads to functional disability and a diminished quality of life. Psychosocial factors can influence the development and chronicity of IBS within a biopsychosocial framework. Standardized hypnotic suggestions directed at the bowel are combined with personalized hypnotherapy, which is effective in both short- and long-term treatment of IBS. It is recommended in European and North American guidelines. It is available on audio cassettes and in digital health applications for mild cases, while severe IBS requires comprehensive, interdisciplinary, personalized treatment that includes individual hypnosis.

Patients undergoing cardiac surgery are particularly vulnerable to psychological trauma and disorders such as depression, anxiety, and post-traumatic stress disorder, which can impair their recovery. Medical hypnosis is an effective means of preventing and treating these psychological problems, leading to better health-related quality of life and cardiovascular outcomes. The article by Tigges-Limmer et al. highlights the effectiveness of medical hypnosis based on clinical experience from a large cardiac center in Germany. The authors advocate training medical hypnosis to cardiac surgery teams to support patient healing. Hypnosis is also frequently used in dentistry, for example, to treat dental anxiety. A study by Benz et al. aimed to determine whether standardized questionnaires on anxiety and personal coping strategies frequently used before dental treatment can influence anxiety levels. The results show that anxiety decreased in the group with the coping questionnaire and increased with the anxiety questionnaire.

## Treatment influencing factors

Extensive research is concerned with the internal and external factors that play a role in hypnosis and hypnotherapy. One of the most important is hypnotizability, i.e. the individual responsiveness to hypnosis and suggestions. Rasch and Cordi investigated the influence of prior experience and type of presentation on the measurement of hypnotizability. They found that hypnotizability is a relatively stable personality trait that shows no major influence of pre-experience or modality of assessment. Di Filippo and Perri take up an old question: Is there a relationship between hypnotizability and attachment style (Peter et al., 2011; Wieder and Terhune, 2019)? They showed, contrary to Peter et al. (2011), that factors of insecure attachment were not associated with the level of hypnotizability, whereas it was associated with variations of consciousness during hypnosis. Siewert et al. tested the hypothesis that outcome expectancy plays a major role in hypnotherapeutic treatments and found that the beneficial effect of, at least, group hypnosis in distressed participants was not associated with outcome expectations. Objective measures of hypnotic trance seem desirable, and devices developed to monitor anesthetic depth might be closer to clinical applications than classical encephalography. Zech, Seeman, et al. report on changes in such an EEG-based index during hypnosis. For a standardized trance-induction, they chose a text used world-wide for hypnotizability testing, namely the Harvard Group Scale of Hypnotic Susceptibility (HGSHS). For higher feasibility of this widely used HGSHS, especially for clinical studies, a shortened version has been developed recently, comprising five instead of 12 test items (Riegel et al., 2021). Zech, Riegel, et al. present and discuss the available first results of this test in comparison to the full version. They describe non-normal score distributions with striking consequences for grouping into low and high hypnotizables. An interesting question is, whether hypnosis can be modulated by neurophysiological interventions. Perri and Di Filippi found evidence that subjects with lower hypnotic responsiveness benefit most from transcranial electrical stimulation (tDCS) of the dorsolateral prefrontal cortex (DLPFC).

## Outlook

Finally, in this Research Topic on Clinical Hypnosis, an outlook to the future seems appropriate. New technical developments like virtual reality and artificial intelligence do not stop at or exclude hypnosis. How virtual reality can be combined with hypnotic induction and interventions is presented by Safy et al. The limited effects reported may reflect the common experience with hypnosis of the pivotal role of personal interaction and therapeutic relationship. Starting from the astounding results of a study on suggestions given during general anesthesia (Nowak et al., 2020) and evidence for perception also in other “disorders of consciousness,” Hansen proposes the application of hypnosis in patients who were previously largely excluded: unconscious patients. He argues that the appropriate language in this case is hypnotic communication, for “Touching the unconscious in the unconscious.” Ever since people have been studying the phenomena and effects of hypnosis, they have been asking questions about its nature. The answers have been very different since 1784 (Franklin et al., 2002) and sometimes very controversial. The debate about whether hypnosis is a special state of consciousness or just mundane, everyday socio-cognitive processes worried hypnosis researchers for decades (Lynn et al., 2015). It is therefore surprising that the theory of predictive coding has not yet been applied to hypnosis. Thus, we are proud to present the contribution of Zahedi et al. as a premiere for this novel theoretical framework.

We hope that with this Research Topic “Clinical Hypnosis,” hypnosis and its therapeutic potential achieve greater recognition and attention, and more patients can benefit from its supplementary use in medicine. Interested readers may find additional information and support from the *International Society of Hypnosis* (ISH; https://www.ishhypnosis.org) or the *European Society of Hypnosis* (ESH; https://www.esh-hypnosis.eu) and their national Constituent Societies, and specialized hypnosis journals (e.g., the International Journal of Clinical and Experimental Hypnosis or the American Journal of Clinical Hypnosis).
